# Radiologic and Clinical Correlates of Long-Term Post-COVID-19 Pulmonary Sequelae

**DOI:** 10.3390/jcm14144874

**Published:** 2025-07-09

**Authors:** Gorkem Durak, Kaan Akin, Okan Cetin, Emre Uysal, Halil Ertugrul Aktas, Ulku Durak, Ahmet Yasin Karkas, Naci Senkal, Hatice Savas, Atadan Tunaci, Alpay Medetalibeyoglu, Ulas Bagci, Sukru Mehmet Erturk

**Affiliations:** 1 Machine & Hybrid Intelligence Lab, Department of Radiology, Northwestern University, Chicago, IL 60611, USA; h.ertugrulaktas@gmail.com (H.E.A.); hatice.savas@northwestern.edu (H.S.); ulas.bagci@northwestern.edu (U.B.); 2 Department of Internal Medicine, Faculty of Medicine, Istanbul University, 34093 Istanbul, Turkey; kaan.akin@istanbul.edu.tr (K.A.); okancetin@istanbul.edu.tr (O.C.); naci.senkal@istanbul.edu.tr (N.S.); alpay.m@istanbul.edu.tr (A.M.); 3 Department of Radiation Oncology, Prof. Dr. Cemil Tascioglu City Hospital, 34384 Istanbul, Turkey; dremreuysal@yahoo.com; 4 Department of Pulmonology, Faculty of Medicine, Istanbul University, 34093 Istanbul, Turkey; ulku.durak@istanbul.edu.tr; 5 Department of Radiology, Faculty of Medicine, Istanbul University, 34093 Istanbul, Turkey; ahmet.karkas@istanbul.edu.tr (A.Y.K.); atatuna@istanbul.edu.tr (A.T.); smerturk@istanbul.edu.tr (S.M.E.)

**Keywords:** long COVID-19, post-acute sequela of COVID-19 (PASC), chronic COVID-19

## Abstract

**Background/Objectives**: The long-term sequelae of COVID-19 pneumonia, particularly the persistence of imaging abnormalities and their relationship to clinical symptoms, remain unclear. While the acute radiologic patterns are well-documented, the transition to chronic pulmonary changes—and their implications for long COVID symptoms—require systematic investigation. **Methods**: Our study included 93 patients with moderate to severe COVID-19 pneumonia who were admitted to Istanbul Medical Faculty Hospital, each having one follow-up CT scan over a ten-month period. Two thoracic radiologists independently calculated semi-quantitative initial chest CT scores to evaluate lung involvement in pneumonia (0–5 per lobe, total score 0–25). Two radiologists and one pulmonologist retrospectively examined the persistence of follow-up imaging findings, interpreting them alongside the relevant clinical and laboratory data. Additionally, in a subcohort (*n* = 46), mid-term (5–7 months) and long-term (≥10 months) scans were compared to assess temporal trajectories. **Results**: Among the 93 patients with long-term follow-up imaging, non-fibrotic changes persisted in 34 scans (36.6%), while fibrotic-like changes were observed in 70 scans (75.3%). The most common persistent non-fibrotic changes were heterogeneous attenuation (29%, *n* = 27) and ground-glass opacities (17.2%, *n* = 16), and the persistent fibrotic-like changes were pleuroparenchymal bands or linear atelectasis (58%, *n* = 54), fine reticulation (52.6%, *n* = 49), and subpleural curvilinear lines (34.4%, *n* = 32). Both persistent non-fibrotic and fibrotic-like changes were statistically correlated with the initial CT score (*p* < 0.001), LDH (*p* < 0.001), and ferritin levels (*p* = 0.008 and *p* = 0.003, respectively). Fatigue (*p* = 0.025) and chest pain (*p* < 0.001) were reported more frequently in patients with persistent non-fibrotic changes, while chest pain (*p* = 0.033) was reported more frequently among those with persistent fibrotic-like changes. Among the 46 patients who underwent both mid- and long-term follow-up imaging, 47.2% of those with non-fibrotic changes (17 out of 36) and 10% of those with fibrotic-like changes (4 out of 40) exhibited regression over the long term. **Conclusions**: Initial imaging and laboratory findings may indicate persistent imaging findings related to long-term sequelae of COVID-19 pneumonia. Many of these persistent imaging abnormalities, particularly non-fibrotic changes seen in the mid-term, tend to lessen over the long term. A correlation exists between persistent imaging findings and clinical outcomes of long COVID-19, underscoring the need for further research.

## 1. Introduction

The impact of large-scale pandemics imposes a heavy burden on the healthcare system, both in the short and long term. As of May 2025, the COVID-19 pandemic has affected over 777 million people and has resulted in more than 7 million deaths worldwide [1]. Unlike the Middle East Respiratory Syndrome (MERS) and Severe Acute Respiratory Syndrome (SARS) epidemics, the vast number of individuals affected by COVID-19 can make even rare long-term complications a significant health burden. These complications, often referred to as long COVID-19, can impact multiple systems, impair physical abilities, lead to abnormal imaging findings, and reduce overall quality of life [2,3]. Long COVID-19 occurs in at least 10% of severe acute cases, with an estimated 65 million people worldwide affected by it [4]. The literature defines the most common persistent clinical symptoms as fatigue, dyspnea, chest pain, muscle pain/myalgia, and cough [5]. However, more than 200 symptoms have been identified, affecting various organ systems, with recovery times exceeding 35 weeks in over 91% of cases [6]. This sustained condition continues to diminish the quality of life, strain healthcare systems, and challenge our fundamental understanding of post-viral respiratory pathophysiology, leaving patients desperately awaiting answers that science has yet to provide [7].

Due to the nature of SARS-CoV-2, the lungs are the organs primarily affected [8,9]. Several mechanisms contribute to lung damage, including direct viral injury, the host immune response, and treatment-related barotrauma, all of which may lead to long-term damage [10]. Persistent lung parenchymal and airway changes are well-known complications of COVID-19, SARS, and MERS, primarily expected to occur in patients recovering from moderate-to-severe acute pneumonia [11,12,13]. However, a standard method for thoroughly understanding persistent imaging findings or how to describe them has not yet been established. Experts suggest that the term ‘fibrosis’ should be avoided initially, as imaging findings usually resolve over time [10]. The term “fibrotic-like changes” refers to scarring-like patterns that are potential precursors of fibrosis and represent findings that are considered likely to be more permanent, serving as an apt description for lung abnormalities following COVID-19 pneumonia [14,15]. In conclusion, there are currently no established clinical or radiological guidelines for follow-up care regarding long-term COVID-19; recommendations typically depend on expert opinion.

Chest computed tomography (CT) remains the gold standard imaging technique for assessing lung parenchyma and provides clear semiology for acute COVID-19 pneumonia [16,17,18,19]. It has played a vital role during the acute phase, particularly in cases with moderate to severe symptoms. It is also commonly used in follow-up imaging for patients with persistent clinical symptoms. Chest CT scans conducted after COVID-19 pneumonia reveal varying rates of persistent abnormal imaging findings at one year, estimated to be between 24% and 54%, including non-fibrotic and fibrotic-like lung changes [20,21,22]. However, the clinical consequences of these imaging abnormalities remain unclear. The uncertainty surrounding these persistent imaging abnormalities constitutes a major clinical concern demanding immediate attention. Despite extensive documentation, we face a persistent uncertainty in linking radiological findings to the long-term clinical symptoms that persistently disable countless COVID-19 survivors. Understanding late complications and persistent symptoms can guide the development of targeted follow-up procedures to reduce long-term morbidity, mortality, unnecessary radiation exposure, and healthcare costs. This retrospective study has two aims: First, to correlate laboratory and clinical data of patients hospitalized with COVID-19 with persistent imaging findings observed after ten months of follow-up. Second, to identify the persistent imaging findings of COVID-19 that may resolve even after five to seven months.

## 2. Materials and Methods

### 2.1. Study Population

**Figure 1:**
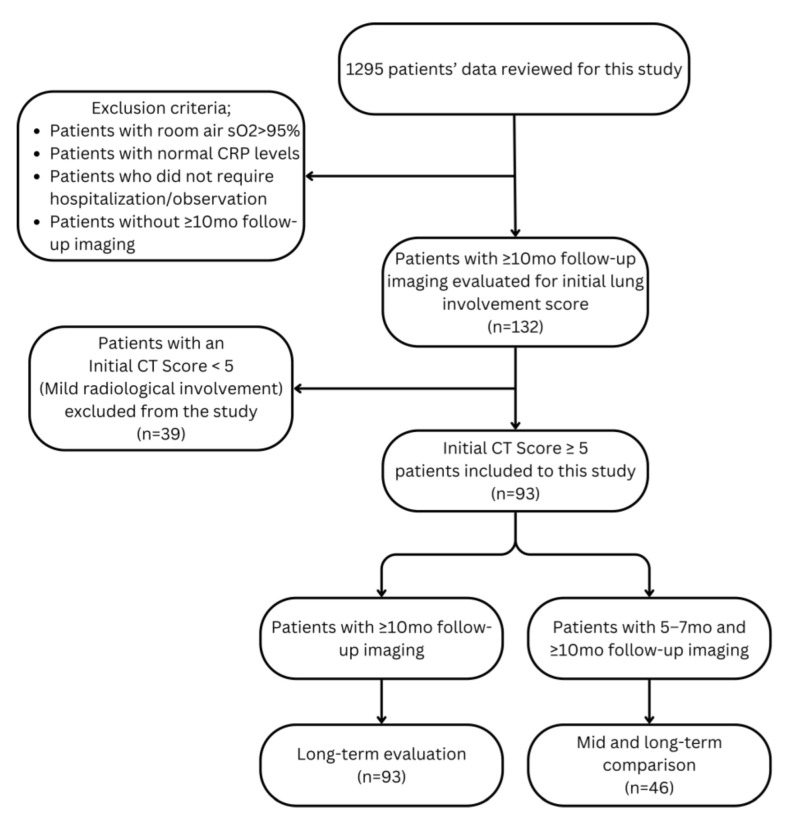
Flowchart for patient selection and evaluation.

Our retrospective, single-center, observational study included 93 patients admitted to Istanbul Medical Faculty Hospital between March 2020 and April 2022, all diagnosed with moderate-to-severe COVID-19 pneumonia, as confirmed and categorized through PCR tests, chest CT findings, and clinical evaluations. Each patient underwent at least two chest CT scans during their initial hospital admission and at a follow-up visit over ten months. Two internists and a pulmonologist reviewed patient histories and reached a consensus on which patients and data to include in the study. Experts assessed monitoring factors such as age, PO_2_ levels, the need for respiratory support, chronic medication use, and other comorbidities. Patients whose room air SpO_2_ levels exceeded 95%, had normal CRP levels, or did not require observation or hospitalization were excluded. An experienced COVID-19 specialist supervised clinical assessments during both admission and follow-up. Following volumetric scoring of the initial CT scans, patients with a lung involvement score below five were classified as having mild parenchymal involvement and were excluded. The scoring method is explained in the ‘CT interpretation’ section. Figure 1 represents the flowchart for patient selection and evaluation.

### 2.2. Clinical and Laboratory Data

The clinical data for the patients included demographic information (age, gender), body mass index (BMI), and laboratory results at the time of diagnosis (lymphocytes, platelets, C-reactive protein (CRP), lactate dehydrogenase (LDH), ferritin, troponin, and D-dimer). It also documented the presence of comorbid conditions such as asthma, hypertension, diabetes mellitus (DM), chronic lung diseases (including chronic obstructive pulmonary disease (COPD) and interstitial lung disease), chronic liver disease, chronic kidney disease, chronic heart conditions (like coronary artery disease and heart failure), solid and hematological cancers, long-term use of steroids or other immunosuppressants, and a history of solid organ or hematopoietic stem cell transplantation. Long-term corticosteroid use is defined as daily or frequent use of systemic corticosteroids for the last three months or longer, or short-term use—typically less than 30 days—of high-dose corticosteroids, generally defined as ≥20 mg/day of prednisone or an equivalent dose. Information about the history of solid or hematological cancer was unavailable for one patient, and details about the transplantation history were lacking for two patients. This missing information was excluded from the statistical analysis. Patients who attended the follow-up were questioned about persistent clinical symptoms such as fatigue, cough, fever, chest pain, muscle pain, and dyspnea.

### 2.3. CT Scan Acquisition

All chest CT scans were conducted in the supine position and during inspiration. Chest CT scans at the diagnosis were obtained using 64-detector Aquilion, Canon (Otawara, Japan), and 16-detector Brilliance, Philips scanners (Amsterdam, The Netherlands), while follow-up scans utilized 64 and 640-detector Aquilion, Canon scanners. The scanning protocol included the following parameters: a tube voltage of 120 kV; a tube current time product ranging from 50 to 150 mAs; a pitch of 0.85–1.4; initial CT scan slice thickness and reconstruction interval of 1–5 mm; and follow-up CT scan slice thickness and reconstruction interval of 1 mm. The intravenous contrast media was not administered.

### 2.4. Initial CT Interpretation

Two radiologists with 6 and 25 years of experience in thoracic radiology reviewed the images and retrospectively calculated semi-quantitative volumetric scores for COVID-19 pneumonia separately based on the initial chest CT scans. They discussed discrepancies in scores, and final decisions were made through consensus. The CT scans were scored using a five-lobe, five-point system to reflect the extent of lobar involvement (0: 0%; 1: <5%; 2: 5–25%; 3: 26–50%; 4: 51–75%; 5: >75%; range 0–5; total score 0–25) [23]. Ground-glass opacities (GGO) without traction bronchiectasis, consolidative opacities, crazy paving, or patterns indicative of organizing pneumonia were regarded as indicators of acute COVID-19 pneumonia and calculated for volumetric scoring. Imaging findings unrelated to COVID-19 pneumonia were also noted and were not included in the volumetric evaluation. Discrepancies were resolved by consensus. When calculating the volumetric CT score, the scan with the highest involvement was selected, and it was assessed whether the patient had multiple CT scans within the first two weeks.

### 2.5. Follow-Up CT Interpretation

**Figure 2:**
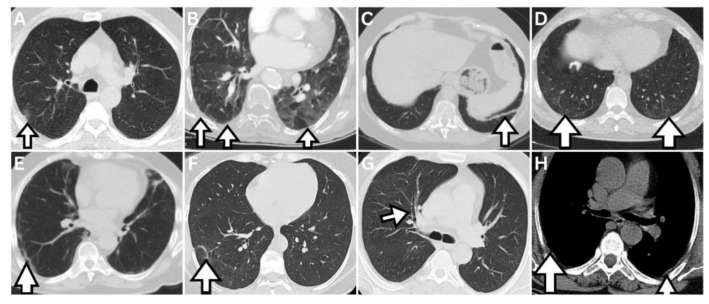
Examples of follow-up persistent imaging findings from our dataset are indicated by arrows. (**A**,**B**) illustrate non-fibrotic changes: (**A**) ground-glass opacity, (**B**) mosaic attenuation. (**C**–**G**) illustrate fibrotic-like changes: (**C**) pleuroparenchymal band, (**D**) subpleural fine reticulation, (**E**) subpleural curvilinear lines, (**F**) parenchymal distortion, (**G**) bronchial changes. (**H**) illustrates pleural thickening in soft tissue window.

The same two radiologists and one pulmonologist retrospectively evaluated the persistence of findings in follow-up imaging without prior knowledge of clinical and laboratory information. The follow-up findings were assessed for new developments or progression compared to the initial imaging while considering pre-existing conditions unrelated to COVID-19. We ensured that the locations of any residual changes were consistent with the initial imaging, as other changes could arise from different situations that may have developed during the interval. We evaluated persistent non-fibrotic changes, including GGO, consolidation, cysts, emphysema, heterogeneous attenuation, and centrilobular nodules, and fibrotic-like changes, including fine reticulation, subpleural curvilinear lines, pleuroparenchymal bands or linear atelectasis, parenchymal distortion, honeycombing, increased extent or severity of traction bronchiectasis/bronchiolectasis, and bronchial/bronchiolar thickening or distortion (hereafter referred to as ‘bronchial changes’), along with pleural thickening. Focal or diffuse heterogeneous attenuation, such as focal air trapping or mosaic attenuation corresponding to areas of initial infiltration, which may indicate small airway damage, was analyzed together. The controversial findings were resolved by consensus. Additionally, we evaluated 46 patients in this cohort with an additional follow-up imaging conducted five to seven months after their diagnosis, referred to as mid-term, to determine if the findings persisted between the follow-up scans. Figure 2 displays examples of persistent follow-up imaging findings from our dataset.

### 2.6. Statistical Analysis

Descriptive statistics were reported as percentages (%) for categorical variables and as medians (range) for continuous variables. Normality of continuous variables was evaluated with histograms, the Kolmogorov–Smirnov test, and the Shapiro–Wilk test. Because no variable met the assumption of normality, between-group comparisons used the two-tailed Mann–Whitney U test. Categorical proportions were compared with the χ^2^ test or, when expected cell counts were <5, Fisher’s exact test. Paired categorical outcomes (mid-term vs. long-term scans) were analyzed with McNemar’s test. Variables showing a univariate association with the outcome at *p* < 0.20 and considered clinically relevant were entered into a multivariable logistic-regression model with forward stepwise selection. Model calibration was assessed with the Hosmer–Lemeshow goodness-of-fit test. All tests were two-sided; *p* < 0.05 was deemed statistically significant. Analyses were performed with IBM SPSS Statistics for Windows, version 26 (IBM Corp., Armonk, NY, USA).

## 3. Results

### 3.1. General Characteristics and Follow-Up Imaging Findings

Of the 93 patients hospitalized between March 2020 and April 2022 for moderate-to-severe COVID-19 pneumonia, the average follow-up period was approximately 18 months (ranging from 10 to 42 months). Ages ranged from 34 to 89, with a median age of 56. Among the patients, 54.8% (*n* = 51) were male, and 45.2% (*n* = 42) were female. The patients’ BMIs ranged from 19.1 to 42.4, with a median value of 27.5. The volumetric scores from CT scans obtained during admission ranged between 5 and 23, with a median value of 10.

The laboratory parameters obtained during admission were as follows: lymphocytes 200–3400 cells/μL (median = 990), platelets 42–495 × 10^3^/μL (median = 198), CRP 6–620 mg/L (median = 50), LDH 143–646 U/L (median = 269), ferritin 28–6000 ng/mL (median = 326), D-dimer 200–18,900 ng/mL (median = 850), and troponin 3–586 ng/L (median = 9). Demographic characteristics, CT scores, and laboratory information are shown in Table 1.

Comorbidities were as follows: hypertension in 47.3% (*n* = 44) of patients, diabetes mellitus (DM) in 30.1% (*n* = 28), long-term steroid use or immunosuppressive status in 12.9% (*n* = 12), cardiovascular disease in 9.7% (*n* = 9), chronic lung disease in 9.7% (*n* = 9), solid cancer in 8.6% (*n* = 8), chronic kidney disease in 8.6% (*n* = 8), asthma in 7.5% (*n* = 7), a history of transplantation in 6.5% (*n* = 6), and hematological cancer in 3.2% (*n* = 3). In our cohort, there was no chronic liver disease. Comorbidities are shown in Table 2.

The persistent non-fibrotic changes included heterogeneous attenuation (29%, *n* = 27) and GGOs (17.2%, *n* = 16). In contrast, the persistent fibrotic-like changes comprised pleuroparenchymal bands or linear atelectasis (58%, *n* = 54), fine reticulation (52.6%, *n* = 49), subpleural curvilinear lines (34.4%, *n* = 32), parenchymal distortion (18.3%, *n* = 17), and bronchial changes (5.4%, *n* = 5). Pleural retraction or thickening was examined as an independent finding and was observed in 6.5% of long-term imaging (*n* = 6). In our study, none of the 93 follow-up scans indicated persistent or newly developed consolidation, pulmonary cysts, emphysema, cavitary nodules, centrilobular nodules, honeycombing, lymphadenopathy, or pleural effusion over the long term.

Over 10 months, fatigue persisted in 30.1% (*n* = 28) of the cohort, dyspnea in 16.1% (*n* = 15), chest pain in 14% (*n* = 13), muscle pain in 11.8% (*n* = 11), and cough in 5.4% (*n* = 5). There were no instances of fever that lasted over ten months in our cohort. Persistent imaging findings and clinical symptoms are shown in Table 3.

### 3.2. Examination and Correlation of Follow-Up Imaging Findings Along with Other Characteristics

Persistent non-fibrotic and fibrotic-like changes were statistically associated with the initial CT score (*p* < 0.001), LDH (*p* < 0.001), and ferritin levels (*p* = 0.008 and *p* = 0.003, respectively). Our study did not find a statistically significant correlation between these persistent imaging findings and age, BMI, lymphocyte counts, platelet counts, CRP, or D-dimer. The long-term characteristics of the persistent non-fibrotic-like and non-fibrotic changes are detailed in Table 4 and Table 5, respectively.

A statistically significant correlation was observed between persistent non-fibrotic changes, chronic kidney disease (*p* = 0.048), and immunosuppression (*p* = 0.027). However, no statistically significant correlation was observed between long-term persistent fibrotic-like changes and comorbidities. The correlation between long-term findings and comorbid conditions is shown in Table 6.

A statistically significant correlation was observed between persistent non-fibrotic changes, fatigue (*p* = 0.025), and chest pain (*p* < 0.001). Additionally, a statistically significant correlation was observed between persistent fibrotic-like changes and chest pain (*p* = 0.033). Our study found no statistical correlation between persistent imaging findings and symptoms such as dyspnea, muscle pain, and cough. The correlation between long-term findings and persistent clinical symptoms is shown in Table 7.

In the multivariable logistic regression analysis (Table 8), baseline chest CT severity score (*p* = 0.020) and serum LDH level (*p* = 0.009) were identified as independent predictors of fibrotic imaging changes at long-term follow-up.

For non-fibrotic imaging changes, independent prognostic factors included baseline chest CT severity score (*p* = 0.010), serum LDH level (*p* = 0.007), and the presence of chest pain at presentation (*p* = 0.049).

### 3.3. Comparison of Mid and Long-Term Persistent Imaging Findings

The comparison of the persistent imaging findings from 46 patients who underwent both mid- and long-term follow-up CT scans revealed that among the 46 patients, non-fibrotic changes were detected in 36 (78.3%) and fibrotic-like changes in 40 (87.0%) during the mid-term. Non-fibrotic changes persisted in 19 (52.8%) of the 36 patients over the long term, while 17 (47.2%) completely regressed. Fibrotic-like changes persisted in 36 (90.0%) of the 40 patients over the long term, while 4 (10.0%) were completely regressed. The GGO observed in 32 scans (69.6%) during the mid-term persisted in 11 scans (23.9%) in the long term. The subpleural curvilinear lines observed in 30 (65.2%) mid-term scans persisted in 16 (35.6%) long-term scans. Fine reticulation was observed in 29 (63.0%) mid-term scans and persisted in 27 (58.7%) long-term scans. Heterogeneous attenuation was observed in 19 scans (41.3%), pleuroparenchymal band/linear atelectasis in 31 scans (67.4%), bronchial changes in 5 scans (10.9%), parenchymal distortion in 11 scans (23.9%), and pleural retraction/thickening in 5 scans (10.9%) during the mid-term scans, with these findings persisting over the long term. Compared to the mid-term scans, no new findings were detected in the long-term scans.

**Figure 3:**
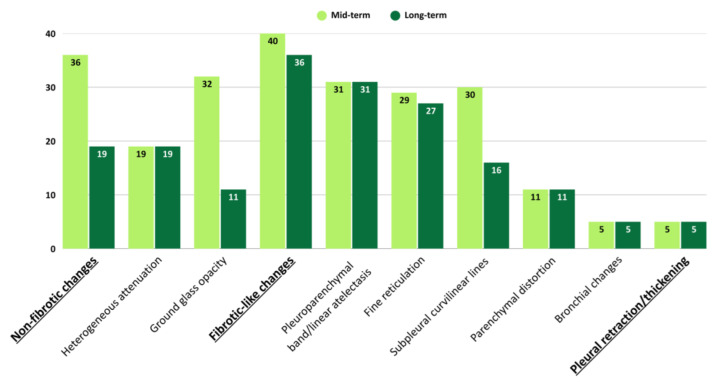
Comparison of persistent imaging findings in patients over mid- and long-term periods.

Non-fibrotic changes, ground-glass opacities (GGO), and subpleural curvilinear lines showed significant differences between mid-term and long-term follow-up (all *p* < 0.001). No significant longitudinal change was observed for heterogeneous attenuation (*p* = 1.000), fibrotic-like changes (*p* = 0.063), pleuro-parenchymal band/linear atelectasis (*p* = 1.000), fine reticulation (*p* = 0.250), parenchymal distortion (*p* = 1.000), bronchial changes (*p* = 1.000), or pleural retraction/thickening (*p* = 1.000). Comparison of persistent imaging findings in patients over mid- and long-term periods is presented in Table 9 and shown in Figure 3.

## 4. Discussion

Our primary results indicate that the long-term prevalence of persistent pulmonary non-fibrotic and fibrotic-like imaging findings in patients with moderate to severe COVID-19 may be as high as 36.6% and 75.3%, respectively. The most common non-fibrotic changes were heterogenous attenuation (29%) and GGO (17.2%). In contrast, the fibrotic-like changes were pleuroparenchymal bands or linear atelectasis (58%), fine reticulation (52.6%), and subpleural curvilinear lines (34.4%). Persistent non-fibrotic and fibrotic-like changes were statistically correlated with the initial CT score (*p* < 0.001), LDH (*p* < 0.001), and ferritin levels (*p* = 0.008, *p* = 0.003, respectively). Fatigue (*p* = 0.025) and chest pain (*p* < 0.001) occurred more frequently in patients with persistent non-fibrotic changes, whereas chest pain (*p* = 0.033) was reported more often in those with persistent fibrotic-like changes. The secondary outcome of our study shows that 47.2% of non-fibrotic changes and 10% of fibrotic-like changes observed in the mid-term have exhibited long-term regression.

Soliman and the COMEBAC study group investigated long-term pulmonary fibrotic lesions in patients hospitalized with COVID-19 pneumonia [24]. The research revealed that among 169 patients who underwent a CT scan at four months, pulmonary fibrotic lesions were identified in 19% (32 out of 169) of patients. Among this group, lesions persisted in 97% (29 out of 30) of patients at nine months and 95% (18 out of 19) at sixteen months. Our cohort included comparatively more severe cases than this study, which explains our higher rates of pulmonary sequelae, as mentioned in our limitations. Additionally, their study focused solely on lesions classified as fibrotic lesions and GGOs, whereas our analysis covers a broader variety of imaging findings. Covering a broader range of findings and including more patients with long-term follow-up imaging provided opportunities to analyze additional characteristics related to long-COVID in our study.

Darcis et al. conducted a study involving patients with confirmed moderate to severe COVID-19. After six months, they analyzed the patients’ CT scans, biological data, and clinical information [25]. This study included 199 individuals, with 47% of patients reporting dyspnea, 32% reporting fatigue, 9% reporting cough, 5% reporting chest pain, and 3% reporting myalgia six months after discharge. Although the frequency of clinical symptoms is similar, these findings have not been correlated with persistent imaging findings. Follow-up chest CT scans revealed a high prevalence of persistent imaging findings, primarily GGOs, affecting 68.9% of the cohort. As highlighted in our study, most of these imaging findings regress, indicating that studies lasting up to six months are insufficient for determining whether there are definite sequelae.

Wahlgren et al. examined 165 hospitalized COVID-19 patients 24 months after their diagnosis, and the majority of these patients (84%, *n* = 139) reported persistent issues affecting their daily lives [26]. Cognitive and sensorimotor problems, along with fatigue, were the most commonly reported persistent symptoms after 24 months. Dyspnea was noted in 124 out of 165 patients, accounting for 76%. In a comprehensive study, Taquet et al. examined EHR data from 273,618 COVID-19 survivors and found that more than one in three patients showed signs of long COVID after a COVID-19 diagnosis, including abnormal breathing or breathing difficulties, fatigue/malaise, chest/throat pain, headache, abdominal symptoms, and myalgia [27]. Both studies indicate that many patients suffer from prolonged symptoms long after the acute phase. These studies emphasize the clinical perspective rather than offering detailed imaging analysis.

In another study by the COMEBAC Study Group, researchers evaluated 478 COVID-19 pneumonia patients hospitalized for new-onset dyspnea and cough four months after hospital discharge [28]. This study also examines the correlation between these symptoms and CT findings, such as fine reticulations, GGO, or fibrotic lesions. New-onset dyspnea was reported in 78 (16.3%), and new-onset cough was reported in 23 (4.8%) of the 478 patients. Similar to our research, no significant difference was found in the type or extent of CT abnormalities between patients with and without new-onset dyspnea. This study spans four months and is not as comprehensive as ours regarding the clinical symptoms and CT findings.

Considering the impacts of higher BMI, diabetes mellitus, chronic kidney disease, and hypertension on hemostasis, the vascular endothelium, and the immune system, it is reasonable to expect that these patients experience more significant damage following their infections. Some studies have demonstrated this correlation between higher BMI, diabetes mellitus, hypertension, and chronic kidney disease with the development and symptoms of long COVID-19 [29,30,31,32,33]. However, research remains insufficient to evaluate these conditions alongside persistent imaging findings. This gap may result in a limited understanding of persistent imaging findings and their correlations with patient clinical presentations in these conditions. Although our study’s dataset is relatively small, it is one of the first in the literature to focus on imaging findings and correlate them with common conditions and clinical symptoms, underscoring the importance of examining these aspects. Despite the association between prolonged clinical symptoms and these accompanying conditions reported in the literature, our study found no statistical correlation with persistent imaging findings.

Our study has several limitations. It was a retrospective, single-center study involving a relatively small cohort size. Additionally, the lack of universally standardized criteria for moderate-to-severe COVID-19 pneumonia classification limits generalizability. While we used consensus-based exclusion criteria (SpO_2_ > 95%, normal CRP levels, no hospitalization required, and CT scores < 5) to focus on patients with more significant disease burden, the absence of clear, universally accepted severity definitions during patient evaluation period makes it challenging to compare our results with other cohorts that may use different severity classification criteria. We exclusively analyzed data from patients with moderate to severe cases, selected based on a combination of imaging and clinical criteria. This intentional selection introduced selection bias; however, focusing on patients at a higher risk for persistent imaging abnormalities addressed the problem more effectively. As a result, the rates of persistent imaging findings we observed were higher than those reported in the broader literature, which often includes milder cases. Some imaging findings we evaluated were relatively mild and localized, such as fine reticulations and pleuroparenchymal bands or linear atelectasis in the fibrotic-like group and GGO in the non-fibrotic group. Including subtle abnormalities may have contributed to the higher observed rate of persistent imaging findings while enabling a more detailed analysis of post-recovery imaging patterns, often underreported in broader, less detailed studies. Due to the retrospective nature of our study, we could not include data on specific COVID-19 pneumonia treatments due to incomplete documentation in medical records, and we were limited to the laboratory parameters that were routinely collected and available in the medical records during the study period. We could not include markers such as IL-6 and procalcitonin, which have been associated with severity in the literature [34], in our analysis because they were not systematically measured for all patients in our cohort.

The prevalence of long COVID-19 stands at 9.5% among individuals who received two doses of the vaccine, compared to 14.6% among those with a single dose or no vaccination, according to the literature [35]. However, vaccination status was not controlled in our cohort, which may introduce variability in the persistence of imaging findings. Despite this limitation, our results provide valuable insights into post-acute imaging patterns independent of vaccination status. We did not gather information on the specific SARS-CoV-2 variants. Since different variants can lead to distinct clinical and imaging manifestations, this may have introduced variability or confounded some findings. Nevertheless, our study captures a real-world range of long COVID-19 imaging outcomes reflective of the studied period. We excluded smoking history from our analysis due to a high rate of missing data. Although smoking is a known confounder in pulmonary outcomes, this exclusion minimized potential bias from incomplete records. Future studies with comprehensive smoking histories will be necessary to clarify its impact on persistent findings. Expiratory high-resolution CT (HRCT) imaging is essential for accurately assessing small airway damage. Since this was not feasible in this retrospective study, only imaging findings detectable in the available images were included in the analysis. Since the retrospective design of the study, we couldn’t assess patient symptoms more objectively; however, we plan to do so in our next study. Finally, our study focused on parenchymal lung abnormalities and did not examine vascular complications, such as pulmonary embolism, due to non-contrast imaging. While this limits our evaluation of thromboembolic events, it highlights the importance of non-contrast imaging in identifying parenchymal sequelae, which are more common among long COVID-19 patients.

## 5. Conclusions

In conclusion, initial imaging along with relevant clinical and laboratory findings may serve as valuable predictors of developing persistent imaging abnormalities related to long COVID-19. Our findings demonstrate a significant statistical correlation between these persistent imaging abnormalities and systemic clinical outcomes, underscoring the need for further investigation to establish causality and clinical implications. A deeper understanding of these imaging patterns and their clinical relevance is crucial for guiding patient management strategies, optimizing follow-up protocols, minimizing unnecessary radiation exposure, and ultimately reducing healthcare costs. Importantly, many persistent imaging findings—particularly non-fibrotic changes—tended to resolve over time, with notable improvement observed between five and seven months after infection. Future research involving larger, multicenter cohorts and longer follow-up periods is essential to clarify these patterns and their long-term clinical impact. Such studies will help refine post-COVID-19 care strategies and enhance outcomes for affected patients.

## Figures and Tables

**Table 1:** Characteristics, initial CT scores, and laboratory information of the patients.

	Median	Min	Max
Age (years)	56	34	89
BMI (kg/m^2^)	27.5	19.1	42.4
Initial CT score (0–25 scale)	10	5	23
Lymphocytes (cells/μL)	990	200	3400
Platelets (×10^3^/μL)	198	42	495
CRP (mg/L)	50	6	620
LDH (U/L)	269	143	646
Ferritin (ng/mL)	326	28	6000
D-dimer (ng/mL)	850	200	18,900
Troponin (ng/L)	10	3	586

**Table 2:** Comorbidities of the patients.

Comorbidities	*n*	%
Hypertension	44	47.3
Diabetes mellitus	28	30.1
Immunosuppression	12	12.9
Chronic lung disease	9	9.7
Cardiovascular disease	9	9.7
Chronic kidney disease	8	8.6
Solid cancer	8	8.6
Asthma	7	7.5
Transplantation	6	6.5
Hematological cancer	3	3.2
Chronic liver disease	0	0.0

**Table 3:** Persistent symptoms and imaging findings over 10 months.

Imaging Findings	*n*	%
**Non-fibrotic changes**		
Heterogeneous attenuation	27	29
GGO	16	17.2
**Fibrotic-like changes**		
Pleuroparenchymal band/linear atelectasis	54	58
Fine reticulation	49	52.6
Subpleural curvilinear lines	32	34.4
Parenchymal distortion	17	18.3
Bronchial changes	5	5.4
**Pleural retraction/thickening**	6	6.5
**Persistent symptoms**	***n***	**%**
Fatigue	28	30.1
Dyspnea	15	16.1
Chest pain	13	14.0
Muscle pain	11	11.8
Cough	5	5.4
Fever	0	0

**Table 4:** Characteristics of long-term persistent fibrotic-like changes.

	Fibrotic-Like Group (*n* = 70)			Remaining Group (*n* = 23)			
	Median	Min	Max	Median	Min	Max	*p*
Age (years)	56	34	82	60	34	89	0.160
BMI (kg/m^2^)	27.9	19.1	42.4	26.7	19.7	38.3	0.373
Initial CT score (0–25 scale)	11	5	23	5	5	18	**<0.001**
Lymphocytes (cells/μL)	970	250	3400	1100	200	2340	0.325
Platelets (×10^3^/μL)	199	42	495	190	52	431	0.415
CRP (mg/L)	59.5	6	620	48	6	320	0.572
LDH (U/L)	303.5	150	646	223	143	465	**<0.001**
Ferritin (ng/mL)	372	28	6000	167	28	1200	**0.003**
D-dimer (ng/mL)	955	240	18,900	580	200	3770	0.102
Troponin (ng/L)	10	3	586	11	3	68	0.629

**Table 5:** Characteristics of long-term persistent non-fibrotic changes.

	Non-Fibrotic Group (*n* = 34)			Remaining Group (*n* = 59)			
	Median	Min	Max	Median	Min	Max	*p*
Age (years)	56.5	44	89	56	34	82	0.798
BMI (kg/m^2^)	28.5	20.5	42.4	27.5	19.1	38.3	0.060
Initial CT score (0–25 scale)	14.5	5	23	8	5	21	**<0.001**
Lymphocytes (cells/μL)	990	250	2600	990	200	3400	0.981
Platelets (×10^3^/μL)	194.5	92	495	200	42	490	0.493
CRP (mg/L)	65	7	620	50	6	359	0.437
LDH (U/L)	342.5	160	646	239	143	447	**<0.001**
Ferritin (ng/mL)	643.5	28	2282	258	28	6000	**0.008**
D-dimer (ng/mL)	945	240	18,900	750	200	4200	0.296
Troponin (ng/L)	15	3	586	9	3	360	**0.035**

**Table 6:** The correlation between long-term findings and comorbid conditions.

	Non-Fibrotic Changes						Fibrotic-Like Changes				
		***n* (0)**	**%**	***n* (1)**	**%**	***p***	***n* (0)**	**%**	***n* (1)**	**%**	***p***
Hypertension	0	34	69.4%	15	30.6%	0.209	12	24.5%	37	75.5%	0.955
	1	25	56.8%	19	43.2%		11	25.0%	33	75.0%	
Asthma	0	56	65.1%	30	34.9%	0.254	21	24.4%	65	75.6%	1.000
	1	3	42.9%	4	57.1%		2	28.6%	5	71.4%	
Solid cancer	0	51	60.7%	33	39.3%	0.250	19	22.6%	65	77.4%	0.104
	1	7	87.5%	1	12.5%		4	50.0%	4	50.0%	
Hematological cancer	0	55	61.8%	34	38.2%	0.293	21	23.6%	68	76.4%	0.153
	1	3	100.0%	0	0.0%		2	66.7%	1	33.3%	
Chronic kidney disease	0	57	67.1%	28	32.9%	**0.048**	23	27.1%	62	72.9%	0.193
	1	2	25.0%	6	75.0%		0	0.0%	8	100.0%	
Chronic lung disease	0	52	61.9%	32	38.1%	0.478	20	23.8%	64	76.2%	0.685
	1	7	77.8%	2	22.2%		3	33.3%	6	66.7%	
Diabetes mellitus	0	42	64.6%	23	35.4%	0.720	16	24.6%	49	75.4%	0.969
	1	17	60.7%	11	39.3%		7	25.0%	21	75.0%	
Cardiovascular disease	0	56	66.7%	28	33.3%	0.069	19	22.6%	65	77.4%	0.218
	1	3	33.3%	6	66.7%		4	44.4%	5	55.6%	
Immunosuppression	0	55	67.9%	26	32.1%	**0.027**	21	25.9%	60	74.1%	0.724
	1	4	33.3%	8	66.7%		2	16.7%	10	83.3%	
Transplantation history	0	57	67.1%	28	32.9%	0.179	22	25.9%	63	74.1%	1.000
	1	2	33.3%	4	66.7%		1	16.7%	5	83.3%	

**Table 7:** The correlation between long-term findings and persistent clinical symptoms.

		Non-Fibrotic Changes					Fibrotic-Like Changes				
		***n* (0)**	**%**	***n* (1)**	**%**	***p***	***n* (0)**	**%**	***n* (1)**	**%**	***p***
Fatigue	0	46	70.8%	19	29.2%	**0.025**	17	26.2%	48	73.8%	0.628
	1	13	46.4%	15	53.6%		6	21.4%	22	78.6%	
Dyspnea	0	52	66.7%	26	33.3%	0.141	22	28.2%	56	71.8%	0.105
	1	7	46.7%	8	53.3%		1	6.7%	14	93.3%	
Chest pain	0	57	71.3%	23	28.8%	**<0.001**	23	28.8%	57	71.3%	**0.033**
	1	2	15.4%	11	84.6%		0	0.0%	13	100.0%	
Muscle pain	0	56	63.6%	32	36.4%	1.000	21	23.9%	67	76.1%	0.594
	1	3	60.0%	2	40.0%		2	40.0%	3	60.0%	
Cough	0	55	67.1%	27	32.9%	0.091	22	26.8%	60	73.2%	0.282
	1	4	36.4%	7	63.6%		1	9.1%	10	90.9%	

**Table 8:** Multivariable logistic regression analysis of factors associated with non-fibrotic and fibrotic imaging changes.

	*p*	OR	95% CI
**Fibrotic-like changes**			
Initial CT score	0.020	1.188	1.028–1.372
LDH	0.009	1.012	1.003–1.020
**Non-fibrotic changes**			
Initial CT score	0.010	1.166	1.038–1.311
LDH	0.007	1.009	1.002–1.015
Chest pain	0.049	5.901	1.011–34.445

OR = Odds Ratio; CI = Confidence Interval.

**Table 9:** Comparison of persistent imaging findings in patients over mid- and long-term periods.

Imaging Findings	Mid-Term		Long-Term	
	***n***	**%**	***n***	**%**
**Non-fibrotic changes**	**36**	**78.3%**	**19**	**41.3%**
Heterogeneous attenuation	19	41.3%	19	41.3%
GGO	32	69.6%	11	23.9%
**Fibrotic-like changes**	**40**	**87.0%**	**36**	**78.3%**
Pleuroparenchymal band/linear atelectasis	31	67.4%	31	67.4%
Fine reticulation	29	63.0%	27	58.7%
Subpleural curvilinear lines	30	65.2%	16	35.6%
Parenchymal distortion	11	23.9%	11	23.9%
Bronchial changes	5	10.9%	5	10.9%
Pleural retraction/thickening	5	10.9%	5	10.9%

## Data Availability

The data supporting the findings of this study are not publicly available due to ethical restrictions. Access to the data can be obtained upon reasonable request to the corresponding author.
